# TLR4‐IN‐C34 protects against acute kidney injury via modulating TLR4/MyD88/NF-κb axis, MAPK, and apoptosis 

**DOI:** 10.22038/IJBMS.2022.67168.14727

**Published:** 2022-11

**Authors:** Hadeer M. Abdelsalam, Manar G. Helal, Nashwa M. Abu-Elsaad

**Affiliations:** 1 Department of Pharmacology and Toxicology, Faculty of Pharmacy, Mansoura University, Mansoura, Egypt; 2 Department of Pharmacology, Faculty of Pharmacy, Horus University, Egypt

**Keywords:** Apoptosis, Isoproterenol, MAPK, MyD88, NF-kappa B, Toll-like receptor

## Abstract

**Objective(s):**

Acute kidney injury (AKI) is a major component of isoproterenol (ISO) induced cardiorenal syndrome. In this study, we investigated the effect of TLR4‐IN‐C34 as a toll-like receptor (TLR)-4 inhibitor on ameliorating ISO-induced AKI and the possible molecular underlying pathways.

**Materials and Methods:**

The study included 4 groups: control group, ISO group (rats received 100 mg/kg ISO in 2 doses 24 hr apart, SC), ISO+C341 and ISO+C343 groups (rats received 1 or 3 mg/kg TLR4‐IN‐C34 respectively twice one hour before each ISO injection, IP).

**Results:**

Obtained results showed that TLR4‐IN‐C34 injection prior to ISO decreased serum creatinine level *(P*<0.05). Renal tissue histopathologic changes were markedly decreased by TLR4‐IN‐C34. Renal relative expression of MAPK and MyD88 mRNA decreased significantly in both ISO+C34_1_ and ISO+C34_3_ groups compared with the ISO group (*P*<0.05). Furthermore, TLR-IN-C34 lowered the inflammatory cytokines IL-8, IL-1β, and IL-12 renal levels (*P*<0.05). Immunostained kidney sections showed a marked decrease in NF-κb positive cells in addition to the apoptotic marker Bax (*P*<0.05) by the two tested doses of TLR4‐IN‐C34. On the other hand, the expression of the antiapoptotic marker Bcl-2 by renal cells was markedly increased.

**Conclusion:**

It can be concluded that TLR4-IN-C34 ameliorates ISO-induced AKI through anti-inflammatory anti-apoptotic effects and modulation of TLR4 signaling pathways.

## Introduction

In response to infections, xenobiotics, a variety of cytokines and environmental stresses, immune cells express toll-like receptors (TLRs) (1). Expression of these receptors is proportional to the severity of organ destruction and disease progression (2).

In the kidneys, many TLRs (TLR1 to 6) are connected with numerous renal sections and contribute to immune system inflammatory responses and renal tissue injury (3). Renal TLR signaling pathways have a clear link with acute kidney damage and have been associated with many cellular processes activated during acute kidney injury (AKI). TLR type 4 (TLR4) is a key molecule in the development of various inflammatory disorders and is best characterized during AKI development. It is considered an upstream modulator of several inflammatory pathways involved in AKI (4).

Inhibition of TLR4 can represent an interesting target to prevent AKI progression into pre-clinical renal diseases. TLR4‐IN‐C34 is a strong and specific inhibitor of TLR4 signaling *in vivo* and *in vitro*. It inhibits TLR4 in enterocytes and macrophages *in vitro* and reduces systemic inflammation in *in vivo* models of endotoxemia and necrotizing enterocolitis (5).

TLR4-IN-C34 introduces a novel family of small compounds with therapeutic potential in illnesses and pathological conditions characterized by overactive TLR4 signaling. It can also help to normalize pathological circumstances or disorders like a faulty electron transport chain or an excess of reactive oxygen species (ROS) (1). Zhang *et al*. (6) demonstrated a potential application of TLR4-IN-C34 in the treatment of central nervous system diseases resulting from neuroinflammation. 

Many studies have found that isoproterenol (ISO)-induced myocardial infarction is accompanied by a cardio-renal syndrome of type 1 (7). Besides, ISO administration increases kidney oxidative stress indicators while decreasing endogenous antioxidants like catalase and glutathione levels. In ISO-treated rats, there is also a significant infiltration of inflammatory cells and marked collagen deposition in the kidneys (8). 

The current study investigates the involvement of the TLR4 signaling pathway in ISO-induced nephropathy. The possible ameliorative effect of TLR4-IN-C34 on AKI development and the proposed mechanisms were inspected.

## Materials and Methods

***Animals***

Adult male Wistar rats (n=34, 150±20 g) were bought from VACSERA (Agouza, Giza, Egypt). Animals were housed 3 weeks before starting the study for accommodation. 

***Chemicals and kits***

DL-Isoproterenol hydrochloride (ISO), 98% crystalline powder, was bought from Alfa Aesar by Thermo Fisher Scientific (Kandel, Germany) (CAS Number: 51-30-9). TLR4-IN-C34 (C34), ≥98% (HPLC), was bought from Sigma Aldrich Chemical Co (St. Louis, MO, USA) (CAS Number: 40592-88-9). Urethane, 99% purity, was purchased from Sigma Aldrich Chemical Company (St. Louis, MO, USA) (CAS Number: 51-79-7).

ELISA Kits for rat interleukin (IL)-8 and IL-1β were purchased from CLOUD-CLONE CORP (USA). ELISA Kit for IL-12 was purchased from lifespan Biosciences (Vienna, Austria). A spectrophotometric kit for creatinine was purchased from Spinreact company (Barcelona, Spain). Super Script IV One-Step RT-PCR kit was purchased from Thermo Fisher Scientific (Kandel, Germany).

***Antibodies***

Rat monoclonal anti-B-cell lymphoma (Bcl)-2 was purchased from Genemed Biotechnologies Inc. (CA, USA). Nuclear factor (NF)-κB p65 rabbit monoclonal antibodies were purchased from ABclonal Science Inc. (Wuhan, China). Anti-Bcl-2 associated X (Bax) rabbit polyclonal antibody was purchased from ServiceBio (Wuhan, China).

***Experimental design***

 Thirty-four rats were divided randomly into four groups as follows: control group (n=10 rats) rats: received normal saline (1 ml/kg, twice, 24 hr apart, SC between ears); ISO group (n=10 rats) received 100 mg/kg ISO (1 ml/kg of 10% w/v solution in normal saline; twice, 24 hr apart, SC between ears) (9), ISO+C34_1_ (n=7 rats) and ISO+C34_3 _(n=7 rats) groups: two groups received 1 or 3 mg/kg TLR4‐IN‐C34 (1 ml/kg of 0.1% or 0.3% w/v solution in water for injection respectively, IP) twice where each dose was injected one hour before each ISO injection. Doses of TLR4‐IN‐C34 were determined through a pilot study and in light of the previous study (5). Table 1 outlines the study’s experimental design.

***Methods***

Twenty-four hours after injecting ISO second dose, rats were anesthetized using urethane (1.8 gm/kg, intraperitoneally) (10). Myocardial infarction was ensured by recording electrocardiograms, increased heart rate, elevated myocardial troponin I, and serum CK-MB (data not shown). Blood samples were collected through retro-orbital sinus, centrifuged, and serum was separated to measure serum creatinine level spectrophotometrically using a ready-to-use kit (11).

The two kidneys were isolated and cut into 3 parts. The first part was snap-frozen in liquid nitrogen and stored at −80 °C until consequent analysis. The second part was cut longitudinally, washed in normal saline, and placed in 10% v/v neutral buffered formalin for paraffin block preparation and further immunohistochemical and histopathological examination. The last part was used for the preparation of kidney homogenate (10% w/v in phosphate buffer pH 7.4).

***Cytokines measurement***

Kidney tissue contents of IL-8, IL-12, and IL-1β were measured in the tissue homogenate using the ELISA technique as directed by the kit manufacturer.

***RNA extraction and quantitative RT-PCR***

Renal expression of mitogen-activated protein kinases (MAPK) and myeloid differentiation primary response (MyD)-88 was measured using qRT-PCR. Kidney parts frozen in liquid nitrogen were ground into powder. Total RNA was then extracted using Direct-zol RNA Miniprep Plus (ZYMO RESEARCH CORP., USA) and its quantity and quality were measured using a Beckman dual spectrophotometer (USA) at 260 nm.

Reverse transcription of extracted RNA was carried out using the SuperScript IV One-Step RT-PCR kit (Thermo Fisher Scientific, Waltham, MA, USA). Designed primers are shown in Table 2 (Vivantis, CA, USA). Briefly, 10 µl of purified RNA was mixed with 40 µl one-step RT-PCR mix containing 25 µl of 2× Platinum™ SuperFi™ RT-PCR Master Mix, 2.5 μl of forward primer (10 μM), 2.5 μl of reverse primer (10 μM), 0.5 µl of SuperScript™ IV RT Mix, and 9.5 µl of nuclease-free water. Components were mixed gently and placed in a pre-heated thermal cycler (Step One Applied Biosystem, Foster City, USA). The following protocols were carried out: cDNA reverse transcription protocol (1 cycle at 55 °C for 10 min) followed by RT Enzyme inactivation protocol (1 cycle at 95 °C for 2 min). Thereafter, cDNA amplification protocol was applied: denaturation (40 cycles at 95 °C for 10 sec), annealing (40 cycles at 55 °C for 10 sec), and extension (40 cycles at 72 °C for 30 sec) followed by the final extension (1 cycle at 72 °C for 5 min). After running RT-PCR cycles, obtained data were stated in Cycle threshold (Ct) for both MAPK and MyD88 and conforming to the housekeeping gene GAPDH. The RQ of each target gene is counted and standardized to housekeeping gene rendering to the calculation of delta-delta Ct (ΔΔCt) (12).

***Histopathology and Immunohistochemistry***

Paraffin wax blocks were processed and 4-μm-thick kidney sections were stained with hematoxylin-eosin stain and examined for tubular injury and interstitial fibrosis. The severity of the renal injury was assessed according to the degree of congestion, interstitial inflammation, and tubular necrosis. The injury was graded semi-quantitatively as follows: score 0 was considered normal, score 1 for mild changes, score 2 for moderate changes, and score 3 for severe changes (13).

Other sections were used for immunohistochemical assessment of NF-κb p-65, BAX, and Bcl-2 protein expression by immunohistochemistry technique. Briefly, after deparaffinization and rehydration antigen retrieval was performed. Sections were incubated with rabbit anti-NF-κB p-65 monoclonal antibodies (A19653), mouse monoclonal anti-Bcl-2 (61-0005), and anti-Bax rabbit polyclonal antibodies (GB11007-1) overnight at 4 °C. After washing, samples were incubated with goat anti-rabbit secondary antibody as a secondary antibody [HRP Goat Anti-Rabbit IgG (H+L) (AS014)] at 1:10000 dilution for 2 hr at room temperature and then visualized with diaminobenzidine. Sections were examined under a light microscope “40x lens” (Leica). Appropriate controls were made to exclude background or non-specific binding (14).

***Statistical analysis***

Data except (histopathologic scores) are expressed as mean ± standard deviation (SD) and analyzed using One-way analysis of variance (ANOVA) test followed by *post-hoc* Tukey-Kramer test. Statistical significance was accepted at (*P*<0.05). Histopathological scores are expressed as median and interquartile range and analyzed using the Kruskal-Wallis test followed by *post-hoc* Dunn’s multiple comparison tests. Graphs were constructed and statistical analysis was carried out using GraphPad Prism V5.01 (GraphPad Software Inc, San Die-go, CA, USA). 

## Results

***Effect on serum creatinine level***

**Figure 1:**
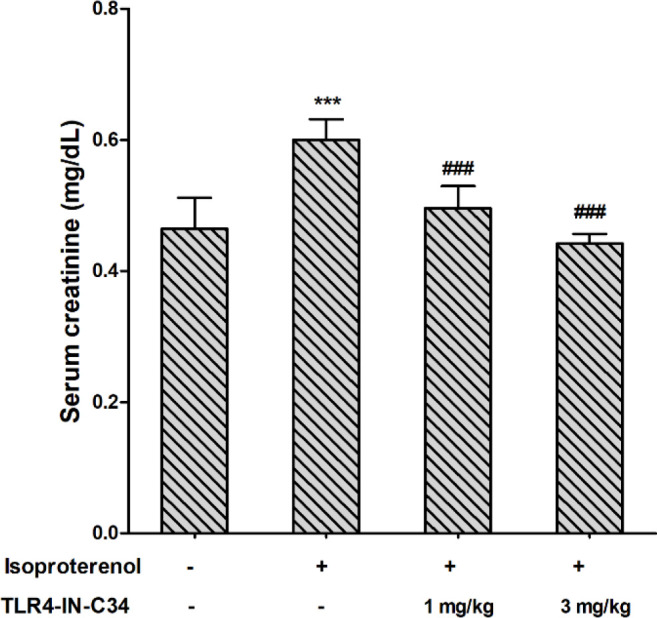
Effect of TLR4-IN-C34 on serum creatinine level in isoproterenol-injected rats. *** *P*<0.001 compared with control group, ### *P*<0.001 compared with isoproterenol group; (n=6/group)

Serum creatinine was significantly (*P*<0.001) elevated by ISO when compared with the control group. In ISO+C34_1_ and ISO+C34_3_ groups, creatinine level was significantly (*P*<0.001) low compared with the ISO group level and became comparable with the control group level (Figure 1). 

***Effect on kidney tissue content of IL-1β, IL-8, and IL-12***

**Figure 2:**
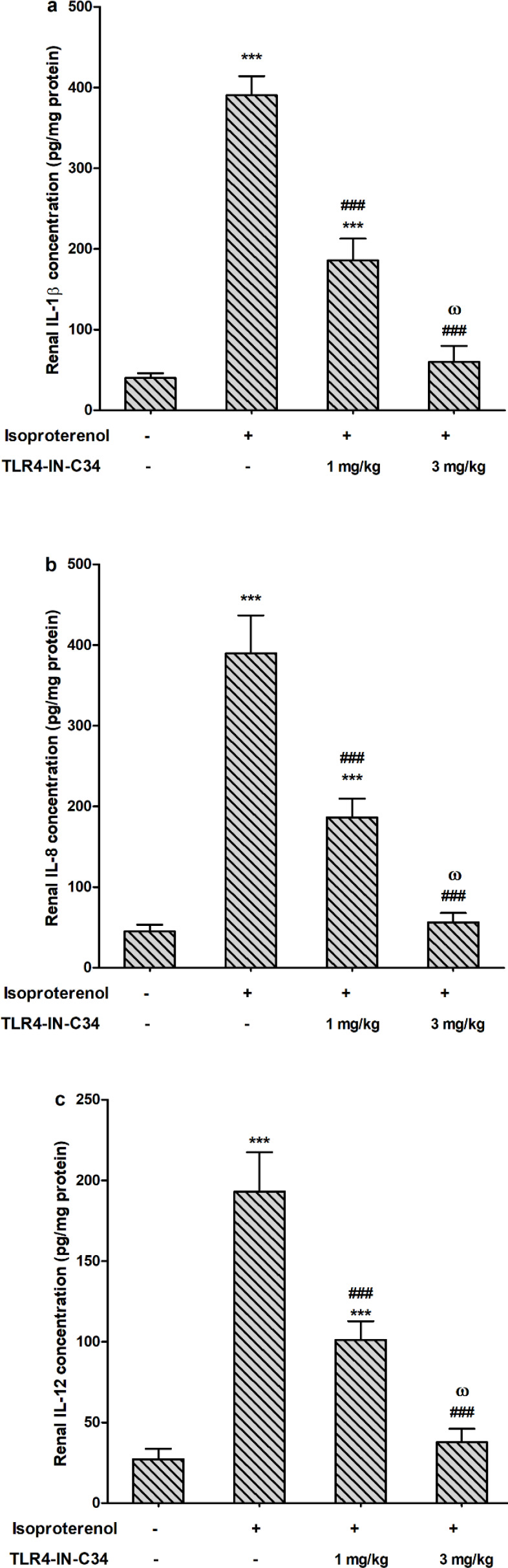
Effect of TLR4-IN-C34 on interleukin (IL)-1β (a), IL-8 (b), and IL-12 (c) levels in isoproterenol injected rats. *** *P*<0.001 compared with control group, ### *P*<0.001 compared with isoproterenol group, and ω *P*<0.001 compared with TLR4-IN-C34 1 mg/kg; (n=6/group)

The group that received ISO alone showed a significantly (*P*<0.001) high renal content of IL-1β compared with the control group. In the ISO-C34_1_ group, renal IL-1β content was significantly (*P*<0.001) decreased when compared with the ISO group but remained significantly (*P*<0.001) higher than the control group renal content. The higher dose of TLR4‐IN‐C34 in the ISO+C34_3_ group significantly (*P*<0.001) decreased IL-1β compared with ISO injection alone and the smaller dose in the ISO-C34_1 _group (Figure 2a).

Injection of ISO alone showed a significantly (*P*<0.001) high renal content of IL-8 compared with the control group. In the ISO-C34_1_ group, renal IL-8 content was significantly (*P*<0.001) decreased when compared with the ISO group. Likewise, C34 (3 mg/kg) decreased IL-8 renal concentration significantly compared with groups that received ISO or ISO+C34_1_ (*P*<0.001). There was no significant difference in IL-8 renal content between the control group and the ISO+C34_3_ group (Figure 2b).

The renal concentration of IL-12 was significantly (*P*<0.001) elevated after ISO injection. Injection of C34 (1 mg/kg) before ISO significantly (*P*<0.001) decreased IL-12 compared with injection of ISO solely but the cytokine content did not reach that of the control group. In the ISO+C34_3_ group, IL-12 concentration was significantly (*P*<0.001) lower than either the ISO group or ISO-C34_1 _group concentrations(Figure 2c). 

***Effect on renal histopathology:***

**Figure 3:**
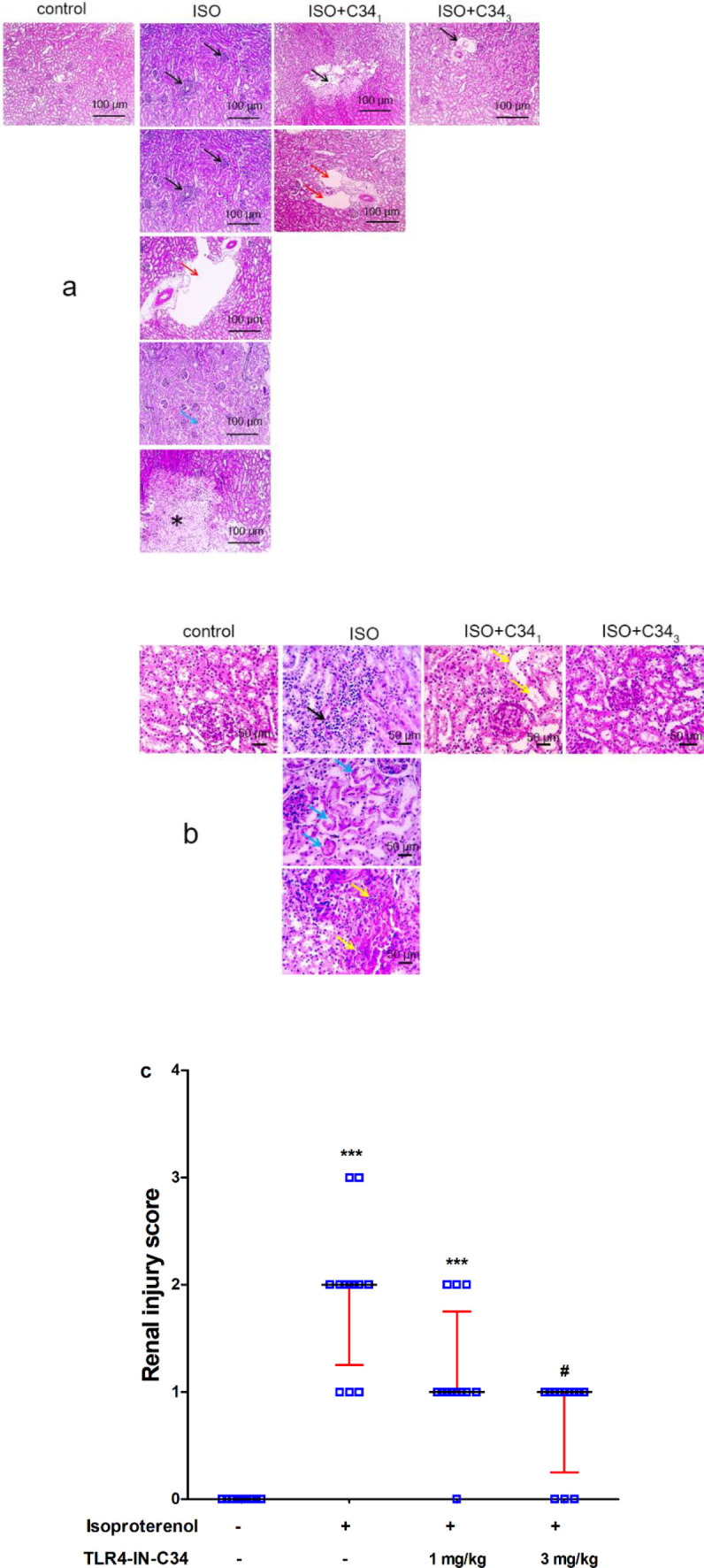
Representative photographs for hematoxylin-eosin-stained kidney sections (a) and (b) showing the effect of TLR4-IN-C34 (1 and 3 mg/kg) on histopathological changes induced by isoproterenol (ISO) as multifocal areas of inflammation (black arrows) in cortex and medulla, perivascular fibrosis (red arrow), desquamation and separation of the tubular epithelium (blue arrows), and focal area tubular necrosis (yellow arrow). (c) Effect of TLR4-IN-C34 on kidney injury score. *** *P*<0.001 compared with control group, # *P*<0.05 compared with isoproterenol group; (n=6/group)

Histopathological examination of kidney sections stained with hematoxylin-eosin stain (Figure 3a and 3b) revealed marked tissue damage in the ISO group. Sections from the ISO group showed multifocal areas of inflammation in the cortex and medulla with perivascular edema. Desquamation and separation of tubular epithelium, tubular necrosis, and extensive fibrosis were observed markedly. In the ISO+C34_1_ group, stained sections showed less renal tissue damage with decreased areas of fibrosis, perivascular edema, and tubular dilation. The higher dose of C34 received by the ISO+C34_3 _group retained normal tubular and glomerular structures with no fibrosis and only mild perivascular edema. Renal injury score in ISO and ISO+C34_1_ was significantly (*P*<0.001) higher than the control group score. A significant (*P*<0.05) decrease in injury score was obtained in the ISO+C34_3_ group compared with the ISO group (Figure 3c). 

***Effect on renal expression of NF-κB p-65, Bax, and Bcl-2***

**Figure 4:**
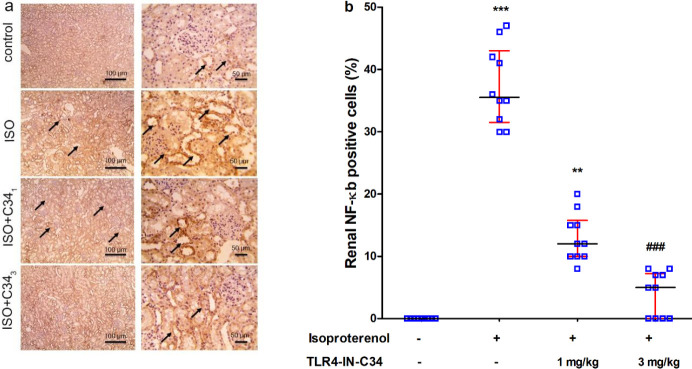
(a) Representative photographs showing the effect of TLR4-IN-C34 (1 and 3 mg/kg) on renal tubular epithelium expression of nuclear factor (NF)-κb (brown stain, black arrows, counter stain: Mayer’s hematoxylin) in kidney sections isolated from rats injected isoproterenol (ISO). (b) Effect of TLR4-IN-C34 on NF-κb expression score. **, *** *P*<0.01, 0.001, respectively compared with control group, ### *P*<0.001 compared with isoproterenol group; (n=6/group)

Immuno-stained sections (Figure 4a) revealed a marked increase in positive brown tubular reaction against NF-κb in the ISO group compared with the control group. The Renal sections stained against Bcl-2 [Figure 6a] showed a strong positive brown tubular reaction in the control group. This reaction was markedly less in the ISO group compared with the control group and was slightly higher in ISO+C34_1_. In the ISO+C34_3 _group, Bcl-2 positive cells were significantly (*P*<0.01) increased compared with the ISO group (Figure 6b). 

***Effect on renal expression of MAPK and MyD88***

Relative expression of MAPK and MyD88 is represented in (Figure 7a). Injection of ISO significantly increased MAPK expression compared with the control group. Low dose TLR4‐IN‐C34 (1 mg/kg) significantly (*P*<0.001) decreased MAPK renal expression when injected before ISO but expression remained significantly (*P*<0.001) high compared with the control group. 

The High dose (3 mg/kg) of TLR4‐IN‐C34 significantly (*P*<0.001) decreased MAPK expression in renal tissue compared with both ISO and C34 low dose (Figure 7b). Additionally, the ISO group showed high (*P*<0.001) expression of MyD88 in the kidney compared with the control group. The group that received ISO+C34_3_ showed a significantly (*P*<0.001, 0.01) low MyD88 expression when compared with the ISO group or ISO+C34_1 _group, respectively (Figure 7c). 

**Table 1:** Schematic representation of the study experimental design

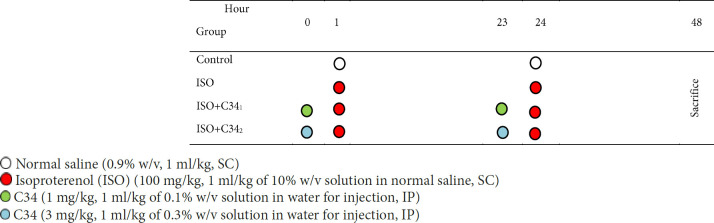

**Table 2:** Primers designed for qRT-PCR of MAPK, MyD88 and GAPDH

	Forward sequence	Reverse sequence
MAPK	5'-ATGGAGAACAACAAAACCTCAGT-3'	5'-TTGCTCCCATGTATGGTCTTTAC-3'
MyD88	5'-AGTTCGGTGGGGTCATGTGTG-3'	5'-CCAGGTATGCACCCAGAGTG-3'
GAPDH	5'-TGGATTTGGACGCATTGGTC-3'	5'-TTTGCACTGGTACGTGTTGAT-3'

MAPK: Mtogen-activated protein kinases; MyD88: Myeloid differentiation factor 88

TLR: Toll-like receptors

TLR: Toll-like receptors

TLR: Toll-like receptors

TLR: Toll-like receptors

**Figure 5:**
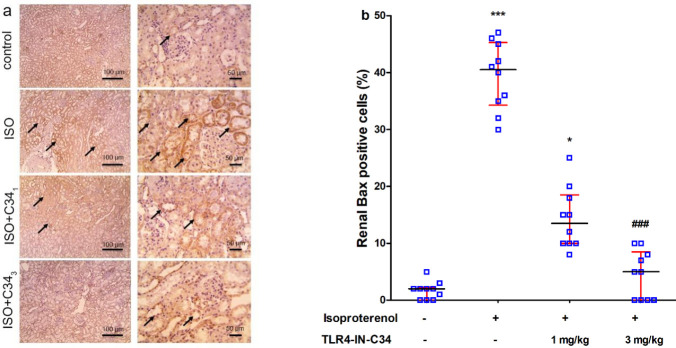
(a) Representative photographs showing the effect of TLR4-IN-C34 (1 and 3 mg/kg) on renal expression of Bax (brown stain, black arrows, counter stain: Mayer’s hematoxylin) in kidney sections isolated from rats injected isoproterenol (ISO). (b) Effect of TLR4-IN-C34 on Bax expression score. *, *** *P*<0.05, 0.001, respectively compared with control group, ### *P*<0.001 compared with isoproterenol group; (n=6/group)

TLR: Toll-like receptors

**Figure 6:**
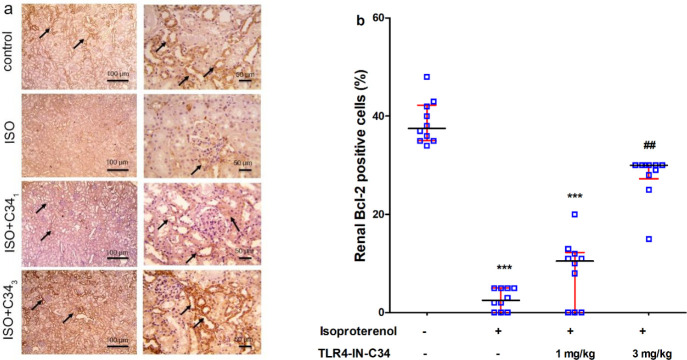
(a) Representative photographs showing the effect of TLR4-IN-C34 (1 and 3 mg/kg) on renal expression of Bcl-2 (brown stain, black arrows, counter stain: Mayer’s hematoxylin) in kidney sections isolated from rats injected isoproterenol (ISO). (b) Effect of TLR4-IN-C34 on Bcl-2 expression score. *** *P*<0.001 compared with control group, ## *P*<0.01 compared with isoproterenol group; (n=6/group)

TLR: Toll-like receptors

**Figure 7:**
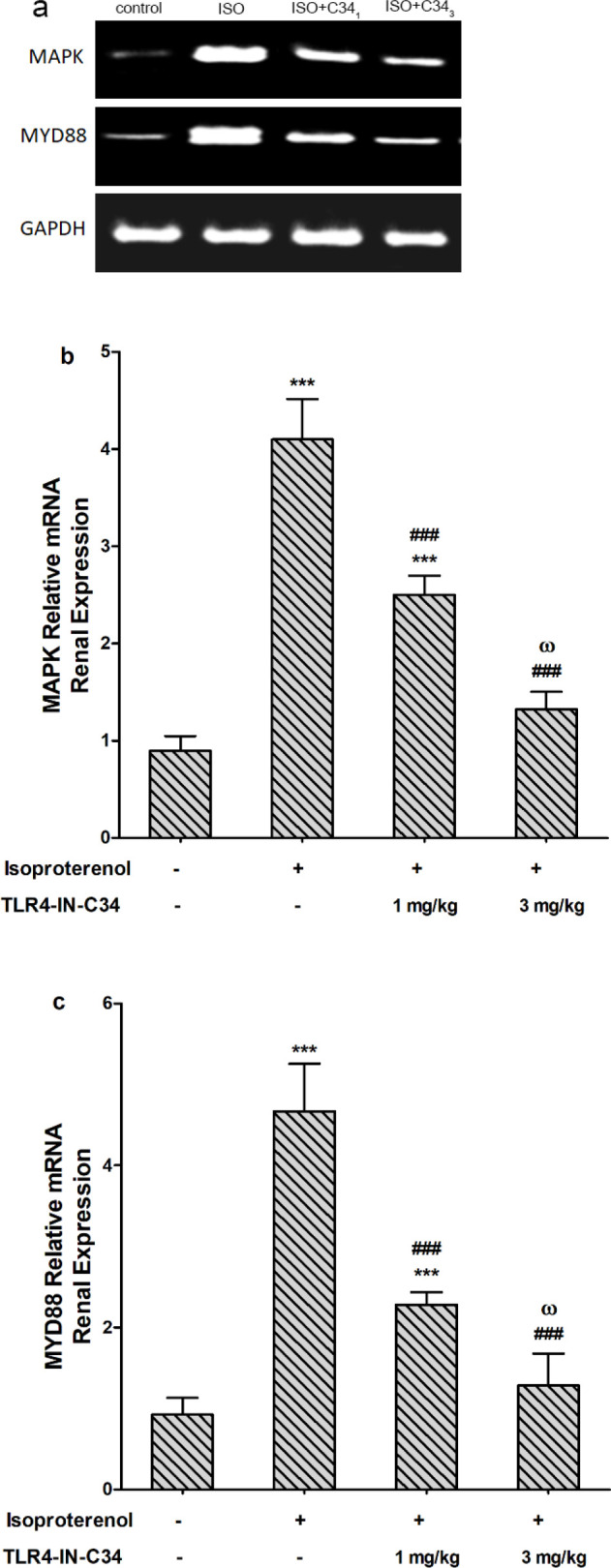
(a) Representative photographs showing mRNA expression bands of renal mitogen-activated protein kinase (MAPK) and myeloid differentiation factor 88 (MyD88). Effect of TLR4-IN-C34 (1 and 3 mg/kg) on (b) MAPK and (c) MyD88 mRNA renal expression (measured by RT-qPCR) in isoproterenol injected rats. *** *P*<0.001 compared with control group, ### *P*<0.001 compared with isoproterenol group, and ω *P*<0.001compared with TLR4-IN-C34 1 mg/kg; (n=6/group)

TLR: Toll-like receptors; MAPK: Mtogen-activated protein kinases

## Discussion

AKI is a progressive renal complication that significantly affects the patient’s life with huge economic burden. Untreated AKI eventually progresses to a chronic form and end-stage renal disease. Increasing evidence shows that TLR4-mediated inflammatory response plays a pivotal role in the pathogenesis of AKI (15).

The current study investigated the role of TLR4 in the management of ISO-induced AKI through the effect of using TLR4-IN-C34, a new selective inhibitor of TLR4. The study involved studying two doses of TLR4-IN-C34 in rats, measurement of serum creatinine level, and examination of renal tissue for histopathological changes. In addition, the effect on cytokines engaged in inflammatory pathways of AKI and apoptotic markers Bax/Bcl2 were evaluated. Targeting TLR4/MyD88/NF-κb and MAPK expression was evaluated as well.

Injection of ISO in large doses resulted in significant damage in renal tissue integrity indicated by histopathologic changes as multifocal areas of inflammation in the renal cortex and medulla, perivascular fibrosis, desquamation, and separation of tubular epithelium. These nephrotoxic effects were accompanied by an increase in creatinine serum level indicating altered renal function. These effects may be explained by activation of the MAPK signaling pathway, reactive oxygen species, and collagen deposition (16). In ISO received group, NF-κb positive cells were markedly increased proposing that ISO increases renal TLR4/NF-κb signaling pathway proteins similar to cardiac tissue as reported by (17).

Toll-like receptor-4 has been implicated in the pathophysiology of a variety of inflammatory illnesses (18). It is a well-known lipopolysaccharide pathogen recognition receptor that plays an important role in the innate immune system. Death-associated molecular patterns (DAMPs) are also recognized by TLR4 and substantially activate its signaling (19). Nephrotoxins result in renal cell damage, and in turn the release of certain DAMPs as HMGB1, triggering the activation of the pattern recognition receptor TLR4 among others and an inflammatory response (20).

Inhibition of TLR4 by TLR4-IN-C34 may halt multiple pathways underlying ISO-induced nephrotoxicity. Promotion of MyD88 is one of the TLR4 signaling pathways that activate NF-κb mediated cytokine release (21). 

Interleukin-8 is a multifunctional chemokine that can proceed as both an anti-inflammatory myokine and a pro-inflammatory cytokine and it has a strong link with AKI in multiple experimental studies (22). It has been applied as a biomarker for AKI following cardiopulmonary surgery (23). Higher content of IL-8 may account for renal parenchymal and tubular damage. This cellular damage results from the chemotactic effect of IL-8 on neutrophils releasing lysosomal enzymes and up-regulating endothelial adhesion molecules (24, 25).

In inflammatory responses, production of IL-8 may be enhanced via the MyD88- independent TLR4 signaling pathway (26). TLR4‐IN‐C34 suppressed IL-8 renal overproduction indicating reduced neutrophils chemotaxis and subsequently renal tissue damage. Similar effects were obtained by other TLR4 inhibitors in some inflammatory diseases (27, 28).

TLR4 is a key molecule that could mediate the NF-κB inflammatory cascade leading to AKI (19). Activation of TLR4/p38MAPK signaling stimulates the inflammatory NF-κB pathway and production of inflammatory cytokines among which are tumor necrosis factor (TNF)-α, IL-1, and IL-6 (30). Our study verified a decrease in NF-κB positive cells and renal levels of IL-1β and IL-6 by TLR4‐IN‐C34. The reduced expression of MAPK renal mRNA may account for the suppression of measured inflammation regulatory cytokines closely associated with renal tissue injury.

Furthermore, TLR4/MyD88/NF-κB downstream signals may be involved in chemically induced AKI. Injection of ISO was accompanied by an increase in mRNA expression of MyD88 in the kidney. These findings were consistent with Yang *et al*. (31) who demonstrated an increase in phosphorylated cardiac NF-κB and TLR4/MyD88 axis activation. The tested inhibitor, TLR4‐IN‐C34, down-regulated MyD88 expression supposing a mechanism for improved renal tissue integrity and kidney function in groups that received the inhibitor.

In injured kidneys, inflammatory reaction induces apoptosis and promotes renal epithelial loss, which is a characteristic of AKI (32). After injury initiation, numerous renal tubular epithelial cells in the nephrons become apoptotic leading to cell necrosis. Damage to the mitochondrial outer membrane also occurs causing increased production of TNF-α, Bax, and caspase, all of which trigger apoptosis. A decreased expression of Bcl-2 with overproduction of Bax is considered a hallmark in apoptosis upstreaming (33).

In addition, various organ damage is highly associated with stimulation of both TLR4 and NF-κB apoptosis-related receptors expression (21). de Ponte *et al*. (34) demonstrated a rise in Bax renal expression upon treatment with ISO signifying the role of β-adrenergic stimulation in inducing intrarenal ROS, endoplasmic reticulum stress, and inflammation. These responses can bring apoptosis in the cortical tubular cells and participate in the development of renal injury. Our study showed similar findings in ISO injected group with a reduction in Bcl-2 expression. Up-regulation of Bcl-2 level while down-regulating that of Bax in groups that received TLR4‐IN‐C34 indicates its ability to inhibit ISO-induced apoptosis and in turn renal injury.

## Conclusion

We believe that TLR4-IN-C34 is a novel therapeutic strategy that decreases ISO-related AKI by modulating TLR4/NF-κB/MAPK/MYD88 signaling thereby attenuating inflammation, oxidative stress, and apoptosis.

## Authors’ Contributions

MGH and NMA Designed the study; HMA Funded the experiment; MH and HA Performed experiments and collected data; HMA and NMA Processed, analyzed, and interpreted data; MGH and NMA Prepared and visualized the manuscript draft; MH and NA Supervised, directed, and managed the study; HA, MH, and NA Approved the final version to be published.

## Data Availability

All data generated or analyzed during this study are included in this published article

## Ethics Approval

All procedures were approved by the institution’s ethics committee for the care and use of animals (approval number: 2021-299). 

## Conflicts of Interest

The authors declare that there are no conflicts of interest.
